# 340B Participation and Safety Net Engagement Among Federally Qualified Health Centers

**DOI:** 10.1001/jamahealthforum.2024.3360

**Published:** 2024-10-04

**Authors:** Elizabeth Watts, Claire McGlave, Nicole Quinones, John P. Bruno, Sayeh Nikpay

**Affiliations:** 1 Division of Health Policy and Management, University of Minnesota School of Public Health, Minneapolis; 2 Cornerstone Research, Chicago, Illinois

## Abstract

**Importance:**

The 340B program provides discounts on outpatient drugs to certain hospitals and federally supported clinics (covered entities) that can be used to generate revenue to fund safety net care. While numerous studies have found no association between 340B and safety net care provision for most hospital covered entities, less is known about whether federally qualified health centers (FQHCs), the largest group of covered entities after hospitals, use the program to enhance safety net care.

**Objective:**

To assess whether a proxy for 340B revenue was associated with increased safety net care provision among FQHCs.

**Design and Setting:**

This descriptive, retrospective cohort study examined care provided from 2005 to 2022 by 1468 FQHCs that submitted to the Health Resources and Services Administration Uniform Data System. FQHC and year-level fixed effects were included, as well as a control for differential Medicaid expansion over time. The data were analyzed between March and December 2023.

**Exposure:**

One-year lagged number of locations registered to dispense or administer 340B-discounted drugs (registered locations), which included child sites, in-house pharmacies, and contract pharmacies in the 340B Outpatient Pharmacy Affairs Database.

**Main outcomes:**

Natural logarithm of patient volume by payer, low-income status, and use of enabling services. Natural logarithm of visits in which low-profit preventive services were provided.

**Results:**

An additional registered location was associated with increased patient volume, especially for uninsured (0.4%; 95% CI, 0.3%-0.5%) and privately insured (0.4%; 95% CI, 0.2%-0.5%) patients and low-income (0.4%; 95% CI, 0.2%-0.6%), unhoused (0.3%; 95% CI, 0.1%-0.5%), and non–English-speaking (0.3%; 95% CI, 0.1%-0.5%) patients. An additional registered location was associated with increased visits with an HIV test (0.7%; 95% CI, 0.4%-0.9%), serum lead test (0.8%; 95% CI, 0.6%-1.1%), seasonal influenza shot (0.4%; 95% CI, 0.3%-0.5%), Papanicolaou smear (0.5%; 95% CI, 0.4%-0.7%), and tobacco cessation counseling (1.0%; 95% CI, 0.5%-1.4%). Across the study period, the average annual increase in locations was 1.5.

**Conclusions and Relevance:**

The results of this cohort study suggest that there are statistically significant increases in the provision of low-profit but high-value preventive services and care to safety net populations (those who lack insurance, have a low income, or require enabling services) and that, like public hospitals, FQHCs might use 340B revenues to enhance safety net care. This finding may inform debates on the 340B program by supporting differential 340B reforms across hospital and nonhospital covered entities.

## Key Points

**Question:**

Are increases in locations registered to dispense or administer 340B-discounted drugs (registered locations) associated with increased safety net care among federally qualified health centers (FQHCs)?

**Findings:**

In this longitudinal cohort study of 1468 FQHCs from 2004 to 2022, increases in registered locations were associated with increases in total, uninsured, and privately insured patient volume, as well as increases in low-income, unhoused, and non–English-speaking populations during the following year. Increases in 340B-registered locations were also associated with increased provision of low-profit but high-value preventive health services, including tobacco cessation counseling and HIV tests.

**Meaning:**

The study results suggest that, similar to recent evidence from public hospitals, FQHCs may be using the 340B program to enhance safety net care.

## Teaser

This cohort study examines whether a proxy for 340B revenue was associated with increased safety net care provision among federally qualified health centers.

## Introduction

The 340B program provides discounts on outpatient drugs to federally supported safety net clinics and certain nonprofit and government-operated hospitals called *covered entities*. The program is economically significant and controversial. Hospitals and clinics that participate in 340B benefit through 2 mechanisms: (1) mitigating financial losses from dispensing free or discounted drugs to patients who cannot pay and (2) generating 340B revenue from charging insurers and patients the full negotiated price for discounted drugs. Although the amount of 340B revenue generated from the spread between insurer reimbursements and discounted 340B prices is unknown, there were $52 billion in discounted 340B purchases in 2021. The list price value of these purchases was $106 billion, implying that the value of 340B revenue could be as high as $54 billion.^1^

Controversy about the program stems from mounting evidence that most hospital covered entities, which account for more than 86% of discounted 340B purchases, do not use the program to meaningfully enhance safety net care.^2,3^ There are no restrictions or guidelines on the use of 340B revenue to allow participating facilities the ability to use revenue in the manner that best facilitates the administration of safety net care. Although the program was originally intended as a smaller program targeted to safety net hospitals and clinics, the 340B program has grown substantially beyond the original targeted group to include nearly half of all US hospitals.^4^ Furthermore, evidence from hospitals suggests that hospitals may use the program to maximize 340B revenue through increased use of high-cost drugs, substantial markups on 340B drugs, expansion of sites registered to administer or dispense 340B drugs to higher-income communities with lower uninsured rates, and possibly increased physician-hospital consolidation.^5,6,7,8^

Although the literature on 340B focuses primarily on hospitals, federally supported clinics comprise 28% of all covered entities, of which federally qualified health centers (FQHCs) are the largest group.^5^ Funded by Section 330 of the Public Health Services Act, FQHCs are required to provide free or reduced-price care to low-income patients. As such, their payer mix differs from other clinicians. On average, 29% of FQHC patients are uninsured, and 42% receive Medicaid coverage.^9^ By contrast, a typical physician practice has less than 6% of patients who are uninsured, and only 17% receive Medicaid coverage.^10^ FQHCs provide primary care and enabling services, such as translation services or health education. They operate traditional clinics as well as alternative delivery sites, such as mobile clinics, school or public housing–based clinics, or seasonal clinics.^11^ While to our knowledge there are no studies on the direct association of safety net engagement with 340B participation, several note the importance of 340B discounts in defraying the costs of providing drugs to patients,^12^ funding medication therapy management programs^13,14^ and disease-specific outreach programs,^13,15,16^ and remaining financially solvent.^17^

As federal and state policymakers consider reforms to the 340B program and manufacturers pursue lawsuits to limit the size of the program, evidence is needed on whether the 340B program may induce the same unintended consequences in federally supported clinics as hospitals.^18,19,20,21^ We address a gap in the research and policy literature on 340B by estimating the association between a proxy for the 340B revenue of FQHCs (the number of locations registered to dispense or administer 340B drugs) and safety net care from 2004 to 2022.

## Methods

We used the Strengthening the Reporting of Observational Studies in Epidemiology (STROBE) cohort checklist when writing this article.^22^ The study was exempt from institutional review board approval as it used exclusively publicly available data that were aggregated to the FQHC year level.

### Data

The analytic dataset was created by linking administrative data from 2 sources: (1) the Health Resources and Services Administration’s (HRSA) Uniform Data System (UDS) and (2) the Office of Pharmacy Affairs Information System (OPAIS) database. The UDS is an annual report on the characteristics of each FQHC’s patient population, services provided, and clinical sites (eg, off-site clinics or temporary care sites).^9^ The OPAIS database, administered by HRSA for the purpose of tracking 340B participation, documents which covered entities are participating, as well as the physical locations where 340B-discounted drugs can be administered or dispensed.^4^ These registered locations include 340B child sites, which are off-site clinics where physicians may administer 340B drugs to patients, or in-house and community pharmacies, called contract pharmacies, where pharmacists dispense 340B drugs to patients. We linked the OPAIS database with the UDS using FQHC grant numbers and names (eMethods 1 in Supplement 1). The resultant FQHC year–level dataset included 1468 unique FQHCs and 21 557 FQHC year observations from 2004 to 2022. The proportion of FQHCs participating in 340B increased from 657 (75%) in 2004 to 1280 (95%) in 2021.

The goal of our analysis was to examine the association of 340B revenue with safety net engagement. However, 340B revenue is not systematically publicly available. For the independent variable, we developed a proxy measure of 340B revenue equal to the count of registered locations (child sites, in-house pharmacies, and contract pharmacies) for each FQHC during each year. We selected this measure as a proxy for 340B revenue because the volume of 340B drugs dispensed, which partially determines 340B revenue, is associated with the number of registered locations. We lagged registered locations by 1 year to reflect the time necessary to generate 340B revenue and make decisions about how to use it to enhance safety net care.

**Figure 1.**
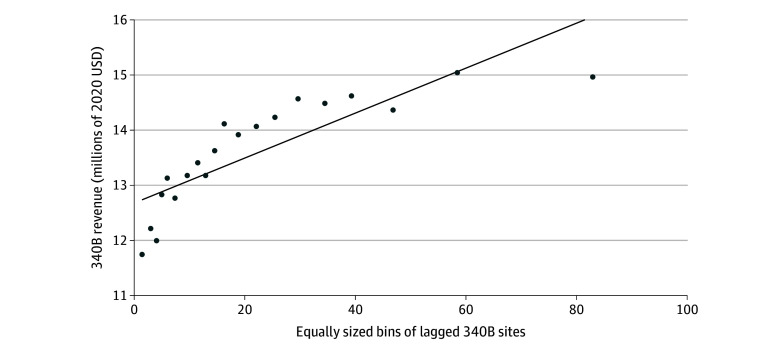
Association of 340B Revenue With Lagged Registered Locations from 2015-2020 Average 340B revenue in millions of dollars against equally sized bins of the lagged number of 340B-registered locations. 340B revenue was winsorized at the 95th percentile and observations with negative values (n = 3) were dropped. The data consist of 829 observations across 317 distinct federally qualified health centers after dropping negative values. Source: Uniform Data System merged to 340B Office of Pharmacy Affairs Database and Federally Qualified Health Center Cost Reports (2015-2020). 2020 $ are reported.

An assumption motivating the use of registered locations as a proxy for 340B revenue is that 340B revenues increase as the number of registered locations increases. While this assumption is supported by anecdotes, we also used a convenience sample of 340B revenue from the 2015 to 2020 FQHC Cost Report of the US Centers for Medicare & Medicaid Services.^23^ The FQHC cost report is an annual report of revenues and costs used to set cost-based reimbursements for FQHCs in Medicaid and Medicare. Because some states require FQHCs to report their costs and adjust them for nonoperating revenue, including the 340B program, we can observe unaudited 340B revenue for a small, nonrandom subset of FQHCs. Figure 1 shows the association between annual 340B revenue and lagged 340B sites. We found that an additional registered location was associated with the log of 340B revenue (1.1%; 95% CI, 0.3%-1.9%) during the following year, implying roughly $17 600 in additional annual revenue per registered location when multiplied by the median reporting FQHC’s annual reported 340B revenue ($1.6 million in 2020). See eTable 1 in Supplement 1 for details on the characteristics of the 340B revenue sample and eMethods 2 in Supplement 1 for the regression specification and results. Although limited, these results suggest that the key assumption of our analysis, that registered locations are associated with 340B revenue, is plausible.

For the dependent variables, we developed measures of the provision of safety net care available in the UDS. They included the count of the number of patients served per year by insurance type (private, Medicare, Medicaid, uninsured) and the count of the number of patients served per year by different characteristics, including family income compared with the federal poverty line, rural residence, and likely need for enabling services,^24^ as indicated by being served in a language other than English and being unhoused. In addition to measuring these patient characteristics, we created dependent variables measuring the number of visits each year in which high community value but low-profit services were delivered. These included HIV tests, seasonal influenza vaccinations, tobacco cessation counseling, Papanicolaou smear tests, and serum lead tests. All outcomes were transformed using the natural log. See eTable 2 in Supplement 1 for a detailed description of the variable creation and eFigure 1 in Supplement 1 for graphs of the unadjusted association between lagged 340B sites and each outcome of interest.

### Statistical Analysis

We used a multivariable ordinary least squares regression to estimate associations between 340B registered locations during the past year and logged safety net engagement measures. The regressions included FQHC-level fixed effects to address time-invariant differences across FQHCs and year-level fixed effects to address secular trends affecting all FQHCs. Due to their focus on the low-income population, FQHCs are sensitive to changes in Medicaid policy. To adjust for changes in care provision that could be associated with expanded Medicaid eligibility under the Affordable Care Act,^25^ we included a time-varying indicator variable set to 1 in the year of and after the state expanded Medicaid. See the full regression specification in eMethods 3 in Supplement 1.

If the 340B program was associated with increased safety net engagement as the program intended, we expect a positive association between lagged registered locations and logged safety net care provision. Standard errors were clustered at the FQHC level to address correlation in unexplained variation across FQHCs over time. Additionally, we estimated results with an alternative measure of registered locations counting only the number of contract pharmacies (rather than in-house pharmacies and child sites) (eMethods 4 in Supplement 1). This sensitivity analysis tests the concern that expansions of the safety net care could reflect expansions of FQHC clinical capacity from increasing clinical sites not associated with the 340B program. By considering only contract pharmacies, we used the variation in registered locations where care was not provided directly by the FQHC.

We present estimated regression coefficients and 95% CIs. We used Stata, version 18 (StataCorp), to perform the analysis and considered results to be statistically significant at the 5% level.

## Results

The average number of 340B registered locations per FQHC increased from 2 in 2004 to 35 in 2021 (eFigure 2 in Supplement 2). On average, FQHCs added 1.5 340B-registered locations per year. We decomposed the locations into 2 groups: those that were part of the covered entity (in-house pharmacies and 340B child sites) and contract pharmacies. Contract pharmacies were associated with most of the growth in prescribing locations, representing 16% of registered locations in 2004 compared with 65% in 2021.

The Table presents the distribution of the safety net care provision measures during the first and last year of the analytic sample. Between 2005 and 2022, the number of FQHCs increased from 906 to 1338. The average patient volume increased by more than 50%, driven primarily by adult patients. The average number of uninsured patients served decreased between 2005 and 2022, but the proportion of patients with all other insurer types increased. There was a larger increase in the number of patients served with incomes greater than 100% of to the federal poverty line compared with patients with incomes less than 100% of the federal poverty line. The number of patients served who likely required enabling services (patients served in a language other than English and unhoused patients) increased. The number of patients from urban zip codes increased more than those from rural zip codes. The average number of HIV tests and tobacco cessation visits per FQHC increased significantly (from a mean of 560 HIV tests and 130 tobacco cessation visits per year to 2683 and 3135, respectively), and we observed moderate increases in influenza vaccinations and lead tests performed. However, the mean number of Papanicolaou tests decreased slightly.

**Table.** Descriptive Statistics of the Dependent Variables, 2005 and 2022

Dependent variable	2005		2022		Difference	Change, %
	No.	Mean (SD)	No.	Mean (SD)		
Patient volume						
Total patients	903	14 664 (15 399)	1338	22 400 (27 504)	7736	53
Patient volume, age 0-17 y	906	5294 (6571)	1338	6421 (9470)	1127	21
Patient volume, age ≥18 y	906	9370 (9307)	1338	15 875 (18 724)	6505	69
Patient volume by payer						
Uninsured	906	5999 (6796)	1338	4198 (6028)	−1802	−30
Public insurance	906	6478 (7882)	1338	14 542 (19 801)	8064	124
Medicare	894	1132 (1310)	1327	2450 (3340)	1318	116
Private insurance	906	2228 (3349)	1338	4486 (6185)	2258	101
Patient volume by income level						
>100% FPL	900	8217 (10 551)	1334	9928 (15 325)	1711	21
101%-200% FPL	906	2458 (3353)	1338	3550 (5136)	1092	44
>200% FPL	867	1040 (2105)	1302	1624 (2876)	584	56
Patients needing enabling services						
Best served in language other than English^a^	1094	3834 (7820)	1267	5827 (11 418)	1994	52
Unhoused	702	349 (1740)	1178	1144 (2532)	795	227
Patient volume by residence						
Patients with rural residence (RUCC 7-9)^b^	901	759 (2220)	1341	1414 (4273)	655	86
Patients with urban residence (RUCC 1-6)^b^	901	4562 (6206)	1341	20 733 (27 393)	16 171	354
Clinical visits, including low-profit, high-value services						
HIV tests	807	560 (1235)	1277	2683 (5386)	2124	380
Tobacco cessation visits^a^	1094	130 (879)	1175	3135 (16 575)	3005	2310
Flu vaccinations^a^	1094	2026 (3791)	1329	3410 (5715)	1384	68
Papanicolaou tests	902	1653 (2354)	1327	1450 (2270)	−204	−12
Serum lead tests^a^	1094	285 (813)	1087	555 (1053)	270	95

Abbreviations: FPL, federal poverty line; RUCC, Rural-Urban Continuum Code.

^a^ Indicates that the earliest year the variable was observed is 2009 (rather than 2005).

^b^ Indicates that the latest year the variable was observed is 2021 (rather than 2022). The difference column represents the difference in means between the latest and earliest year observed. The percentage change was calculated compared with the earliest year.

**Figure 2.**
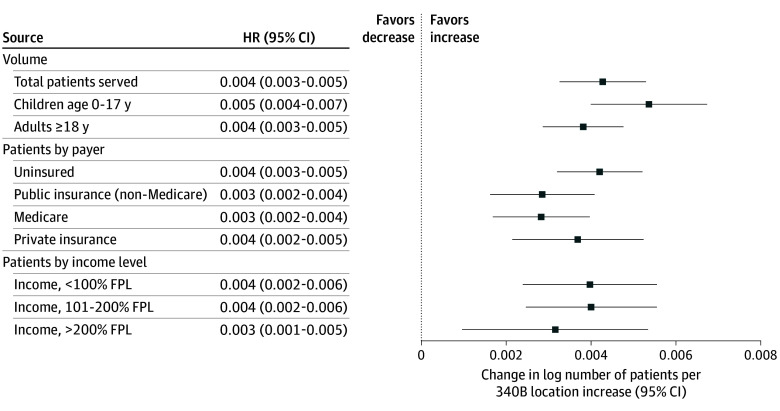
Association of Lagged Registered Locations With Volume and Payer Mix from 2005 to 2022 The horizontal bars show the magnitude and 95% CIs of associations between lagged 340B locations (sum of on-site locations and contract pharmacies) and outcomes of interest listed. All outcomes are log-transformed. Example interpretation: a 1-location increase is associated with an increase in the log number of patients of 0.004, or approximately 0.4%, the next year. Source: Uniform Data System (2005-2022) linked to Office of Pharmacy Affairs Information System (2023). HR indicates hazard ratio.

Figure 2 presents estimated associations between the lagged number of registered locations and logged measures of safety net engagement associated with patient characteristics. The number of registered locations in the previous year was associated with increases in total patients served (0.4%; 95% CI, 0.3%-0.5%), with slightly larger associations for uninsured (0.4%; 95% CI, 0.3%-0.5%) and privately insured patient volume (0.4%; 95% CI, 0.2%-0.5%) than public (0.3%; 95% CI, 0.2%-0.4%) and Medicare volume (0.3%; 95% CI, 0.2%-0.4%). Increases in patient volume were slightly higher among patients with family incomes less than 100% of the federal poverty level (0.4%; 95% CI, 0.2%-0.6%) than among patients with higher family income compared with poverty levels (0.3%; 95% CI, 0.1%-0.5%). Multiplied by the typical number of registered locations gained in a year (1.5), these estimates implied a 0.6% increase in uninsured, privately insured, and low-income patients, as well as a 0.5% increase in Medicaid-covered and Medicare-covered patients.

**Figure 3.**
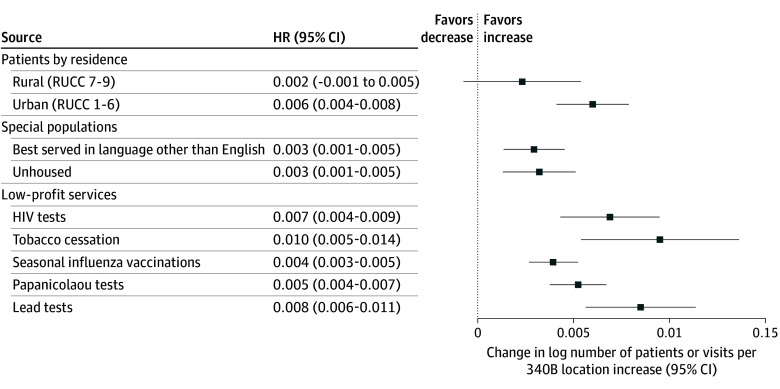
Association of Lagged Registered Locations With Patient Populations and Services from 2005 to 2022 The horizontal bars show the magnitude and 95% CIs of associations between lagged 340B locations (sum of on-site locations and contract pharmacies) and outcomes of interest. All outcomes are log-transformed. Example interpretation: a 1-location increase is associated with an increase in the log number of unhoused patients of 0.003, or approximately 0.3%, the next year. Source: Uniform Data System (2005-2022) linked to 340B Outpatient Pharmacy Information System (2023). HR indicates hazard ratio; RUCC, Rural-Urban Continuum Code.

Figure 3 presents estimates of the association between the lagged number of registered locations and low-profit services provided, or numbers of patients served who were more likely to require enabling services. We found that a 1-location increase was associated with an increase in visits in which an HIV test (0.7%; 95% CI, 0.4%-0.9%), serum lead test (0.8%; 95% CI, 0.6%-1.1%), tobacco cessation counseling (1.0%; 95% CI, 0.5%-1.4%), seasonal influenza vaccination (0.4%; 95% CI, 0.3%-0.5%), or Papanicolaou test (0.5%; 95% CI, 0.4%-0.7%) was performed the following year. An additional location was also associated with increases in visits for patients served in a language other than English (0.3%; 95% CI, 0.1%-0.5%) and visits for patients who were unhoused (0.3%; 95% CI, 0.1%-0.5%). There was no statistically significant association between 340B locations and the number of patients served from rural zip codes. Multiplied by the typical number of 340B locations gained in a year (1.5), these estimates implied a 0.6% and 1.5% increase in low-profit services. See eTables 3 and 4 in Supplement 1 for tabular results for Figure 2 and Figure 3, respectively.)

As a sensitivity check, we reestimated the results without the log transformation of the dependent variable and demonstrated that our findings were qualitatively similar (eTable 5 in Supplement 1). Additionally, results using only lagged 340B contract pharmacies as the explanatory variable were nearly identical to the primary results (eMethods 4 in Supplement 1).

## Discussion

Debates about the 340B program have focused on evidence from hospitals, which suggests that revenues are not directed to the provision of safety net care,^2,3,26^ with the exception of public hospitals.^27^ However, FQHCs also participate in the program and may face different incentives and constraints than hospitals. To our knowledge, these results provide the first systematic analysis of the association between 340B participation and FQHC safety net engagement and suggest that, unlike hospitals, 340B may be associated with enhanced care for key populations that rely on the safety net. Specifically, FQHCs expand care disproportionately to patients who have lower incomes, are served in languages other than English, or are unhoused. The similarly sized increase in uninsured and privately insured patients suggests that FQHCs do not make efforts to selectively treat patients for whom the FQHC will receive insurer reimbursement and thus generate 340B revenue. Furthermore, we found an increase in uninsured patients even as uninsured volumes were falling across health centers over time. Additionally, our finding that 340B participation was associated with increased provision of low-profit, high community value preventive services contrasts sharply with recent studies showing no increase in the provision of low-profit services for certain 340B hospitals.^3,28^

Our results for FQHCs make sense given the differences in payer mix across FQHCs and hospitals and the requirements that FQHCs provide free and reduced-price care to patient populations. With community oversight, location in low-income communities, and the requirement to provide sliding-fee scale care to patients, FQHCs have limited scope to maximize the 340B discounts through acquisition of drug intensive service lines or by shifting across categories of community benefit.^28^ Similarly, FQHCs are less able to generate revenue from insurer reimbursements than hospitals due to their payer mix. Among inpatient hospitals in the US, the average Medicare and private insurance fraction of discharges is 74%, while the remaining 26% were either uninsured or covered by Medicaid. In contrast, the patient populations of FQHCs are on average 36% Medicare-covered or privately insured, with the remaining 63% receiving Medicaid or uninsured (based on reporting in the UDS). FQHCs are also limited in the potential to generate 340B revenue from Medicaid patients. Several states, including Arizona, reimburse covered entities for drugs dispensed to Medicaid patients at the discounted 340B price.^29^

In addition to providing what to our knowledge are the first estimates of the association between a proxy for 340B revenue and safety net engagement for FQHCs, our estimates add to the literature by providing support for using the number of 340B-registered locations as a proxy for 340B revenue, as other studies have done.^30^ Our estimation of associations with an admittedly small, selected sample of FQHCs suggest that an additional registered location brings approximately $17 600 of 340B revenue per year. To approximate the financial value of 340B participation for the typical FQHC, we multiplied the average revenue per site by the median number of locations in 2020 (19 locations). Our results implied $334 400 in 340B revenue per year ($17 600 for 19 locations). For FQHCs in the top 10% by number of registered locations (at least 82 locations), our results implied an annual revenue of $1.4 million per year ($17 600 for 82 locations). These results aligned with anecdotal reports on FQHC revenue. New York State reported an average of $4.5 million in annual revenue for each FQHC in the state.^31,32^ The national annual 340B revenue to FQHCs is estimated to be between $8 billion and $12 billion.^33^ Compared with other sources of income for FQHCs, 340B revenue is important. For comparison, the median FQHC revenue from federal, state, and local grants in 2020 in the UDS was $5 200 000. Even in a year in which FQHCs received additional funding associated with the COVID-19 pandemic, 340B revenue exceeded 25% of grant funds for the top 10% of FQHCs.

FQHCs are heterogeneous entities with distinct goals and patient populations, which complicates efforts to measure the effect of 340B, but comparing marginal (per location) increases in 340B revenue and service provision is important for policy conversations. Among FQHCs that reported 340B revenue, we estimated that the median entity would serve an additional 10 uninsured patients for each 340B location gained during the previous year (eMethods 5 in Supplement 1). Based on HRSA data for the average cost per patient in 2020 ($1157), it would cost the FQHC $11 600 per year, which is less than our estimate of the revenue per location gained ($17 600) but is not insignificant.^34^ Our results also showed that 340B locations were associated with small increases in serving patients needing enabling services. The median FQHC would serve 1 more unhoused patient and 4 more patients needing translation services per location. The cost of serving unhoused patients may be twice that of serving housed patients, and some of the costs incurred are likely to be uncompensated or undercompensated by insurers.^35^ As for the low-profit services included in our outcomes, the median FQHC would provide an additional 4 HIV tests, 2 tobacco cessation visits, 7 flu vaccinations, 3 Papanicolaou tests, and 1 lead test per 340B location added, for an estimated total value of $2000 based on Medicare reimbursement rates, some of which may be uncompensated. Since the average FQHC added 1.5 340B locations per year, increases in safety net–reliant patients could total 15 uninsured patients, 1 unhoused patient, and 6 patients needing translation services. Extrapolating to all FQHCs (n = 1342 in 2020), increased 340B engagement would be associated with 20 100 more uninsured patients ($23 million in uncompensated care), 1300 more unhoused patients, and 8000 more patients needing translation services served annually. Although these estimates do not represent any specific FQHC, they help contextualize the value of 340B revenue, which can be used to close the gap between other revenue sources and the cost of serving patients with complex needs.

Our results also potentially have implications for policy debates on the 340B program. The first concerns the growing use of contract pharmacies. Since 2020, many pharmaceutical manufacturers have stopped providing discounts through all but 1 contract pharmacy per entity in response to the rapid growth of the 340B program.^36^ Our independent variable (340B registered locations) was largely associated with the growth of contract pharmacies in later years. Our results suggest that these restrictions may inhibit the ability of FQHCs to generate 340B revenue and potentially force them to scale back safety net care. A recent survey by the National Association of Community Health Centers found that most FQHCs reported that at least 10% of patients go without medications when 340B discounts are limited.^37^ Manufacturer restrictions could be particularly effective for expensive drugs that tend to be used by safety net–reliant populations, such as the hepatitis C drug sofosbuvir (Sovaldi; Gilead), which costs $84 000 per course of treatment.^38^ Only about half of manufacturer letters restricting contract pharmacy arrangements have exemptions for FQHCs.^39^

Second, our results raise questions about how well the 340B program’s eligibility criteria target resources to safety net hospitals and clinics. FQHCs, which provide free and reduced-price care to 1 in 11 US individuals, may align with the original intent of the program more than the typical participating hospital, yet FQHCs account for only 5.2% of total discounted purchases through 340B.^1,40^ In comparison, disproportionate share hospitals claim more than 70% of discounted purchases through hospitals.^1^ Given the importance of Medicaid to many FQHCs, policymakers should also consider how Medicaid reimbursement rules are associated with the ability of FQHCs to stretch scarce federal resources as the program intended.^41^

Third, our study highlights the importance of transparency in the 340B program. Studies have been limited by the unavailability of uniformly reported and systematically available public data on 340B revenues and costs. Although hospitals have fought to prevent such transparency efforts, greater transparency may help core safety net health care networks, such as FQHCs, demonstrate the role of 340B revenues in expanding access to care for safety net–reliant patients.^42^ New reporting requirements in Minnesota and Maine will help policy makers, advocates, and the public understand the size of the 340B subsidy to FQHCs.^20,43^

### Limitations

This study had several limitations. First, while we can estimate associations between additional locations that generate 340B revenue, we were only able to observe 340B revenue for a small, selected sample of FQHCs. Thus, our results pertaining to the association between registered locations and 340B revenue should be viewed as a validity check of the research design rather than definitive estimates of the program’s benefits. Second, because all FQHCs have been eligible for 340B since the start, we have no quasiexperimental study design to compare FQHCs that do and do not participate in 340B, so we cannot account fully for unobserved confounding factors that were associated with the lagged number of registered locations and safety net engagement. Therefore, we treated our estimates as associations rather than causal effects of the program (see eMethods 6 in Supplement 1 for additional discussion). Despite this limitation, we believe our results provide helpful information for policy debates. Third, we are only able to observe an abbreviated set of safety net engagement measures for FQHCs (payer mix, special populations, and selected services), but these measures may be insufficient to capture all activities that FQHCs pursue to address upstream determinants of health.^44^ Furthermore, we were only able to observe safety net outcomes and not mechanisms through which 340B revenue might be associated with increased safety net care, such as hiring additional staff, opening new sites, and providing free drugs to patients. Finally, our analysis did not address differences in Medicaid reimbursement methods for 340B drugs across states and time. For example, FQHCs in states that use cost-based reimbursement or carve out 340B from the Medicaid program would benefit less from increasing 340B participation than others, biasing our estimates downward.^45^ Lastly, our analysis cannot speak to the efficiency of using 340B to finance safety net care. The question of whether 340B is an effective mechanism for financing safety net care is beyond the scope of this article.

## Conclusions

In this cohort study, we found that increases in 340B registered locations, and therefore 340B revenue, were associated with increases in provision of care at FQHCs, especially to populations relying on the safety net (low-income, non–English-speaking, uninsured, and unhoused individuals). Our measure of 340B registered locations included on-site locations where 340B discounted drugs could be administered (in-house pharmacies and child sites) or dispensed (contract pharmacies) for the purposes of generating 340B revenue. FQHCs also provided more low-profit, high community value preventive services after the number of registered locations increased. These results suggest that FQHCs may use the program differently than many hospitals participating in the program, which have not been shown to increase safety net care after participating.

## Notes

Supplement 1. eMethods 1. Details on OPAIS and UDS cleaning and linkages eMethods 2. Association between Lagged 340B Locations and 340B Revenue eMethods 3. Regression specification eMethods 4. Robustness check using contract pharmacies as the independent variable eMethods 5. Cost of Additional Service Provision eMethods 6. Alternative specifications eTable 1. Characteristics of FQHCs reporting 340B Revenue eTable 2. Detailed Description of Independent and Dependent Variable Creation eTable 3. Regression Results for Figure 2 eTable 4. Regression results for Figure 3 eTable 5. Regression Results with outcomes in levels (not log transformed) eFigure 1. Unadjusted Relationship between 340B Locations and Outcomes eFigure 2. Average Number of 340B Registered Locations per FQHC, 2004-2021

Supplement 2. Data sharing statement

## References

[1] Fein AJ. The 340B program reached $54 billion in 2022—up 22% vs. 2021. Accessed February 19, 2024. https://www.drugchannels.net/2023/09/exclusive-340b-program-reached-54.html

[2] Desai S, McWilliams JM. Consequences of the 340B Drug Pricing Program. N Engl J Med. 2018;378(6):539-548. doi:10.1056/NEJMsa1706475 29365282 PMC6073067

[3] Nikpay SS, Buntin MB, Conti RM. Relationship between initiation of 340B participation and hospital safety-net engagement. Health Serv Res. 2020;55(2):157-169. doi:10.1111/1475-6773.13278 32187392 PMC7080377

[4] Health Resources & Services Administration, Office of Pharmacy Affairs. 340B covered entity data. hrsa.gov. Accessed March 8, 2024. https://340bopais.hrsa.gov/coveredentitysearch?AspxAutoDetectCookieSupport=1

[5] Health Resources & Services Administration. Search covered entities. Accessed March 8, 2024. https://340bopais.hrsa.gov/CoveredEntitySearch/000072748

[6] Chang J, Karaca-Mandic P, Nikpay S, Jeffery MM. Association between new 340B program participation and commercial insurance spending on outpatient biologic oncology drugs. JAMA Health Forum. 2023;4(6):e231485. doi:10.1001/jamahealthforum.2023.1485 37351874 PMC10290244

[7] Robinson JC, Whaley C, Dhruva SS. Hospital prices for physician-administered drugs for patients with private insurance. N Engl J Med. 2024;390(4):338-345. doi:10.1056/NEJMsa2306609 38265645

[8] Liu ITT, Wang J, Sarpatwari A, Kesselheim AS, Feldman WB. Commercial markups on pediatric oncology drugs at 340B pediatric hospitals. Pediatr Blood Cancer. 2024;71(9):e31158. doi:10.1002/pbc.31158 38970222 PMC11407276

[9] Health Resources & Services Administration. Health center program uniform data system data. Accessed March 4, 2024. https://data.hrsa.gov/tools/data-reporting

[10] Gillis KD. Physicians’ patient mix—a snapshot from the 2016 Benchmark Survey and changes associated with the ACA. Accessed February 16, 2024. https://www.ama-assn.org/sites/ama-assn.org/files/corp/media-browser/public/health-policy/PRP-2017-physician-benchmark-survey-patient-mix.pdf

[11] Bureau of Primary Health Care. Health center program compliance manual. Accessed February 20, 2024. https://bphc.hrsa.gov/compliance/compliance-manual

[12] National Association of Community Health Centers. Health Centers and the 340B Drug Discount Pricing Program: Increasing Access to Essential Medications and Services to Communities in Need (Fact Sheet #0614). Published online 2014.

[13] Rodis JL, Sevin A, Awad MH, . Improving chronic disease outcomes through medication therapy management in federally qualified health centers. J Prim Care Community Health. 2017;8(4):324-331. doi:10.1177/2150131917701797 28381095 PMC5932724

[14] Rodis JL, Irwin AN, Valentino AS, Erdmann AM. Pharmacist care in federally qualified health centers: a narrative review. J Am College Clin Pharm. Published online August 25, 2022. doi:10.1002/jac5.1696

[15] Bartholomew TS, Grosgebauer K, Huynh K, Cos T. Integration of hepatitis C treatment in a primary care federally qualified health center; Philadelphia, Pennsylvania, 2015-2017. Infect Dis (Auckl). 2019;12:1178633719841381. doi:10.1177/1178633719841381 31065216 PMC6488784

[16] Ventura LM, Beatty KE, Khoury AJ, . Contraceptive access at federally qualified health centers during the South Carolina choose well initiative: a qualitative analysis of staff perceptions and experiences. Womens Health Rep (New Rochelle). 2021;2(1):608-620. doi:10.1089/whr.2021.0060 35141709 PMC8820399

[17] Traynor K. In rural Iowa, 340B program funds critical services. Am J Health Syst Pharm. 2018;75(6):334-336. doi:10.2146/news180017 29523529

[18] Cornerstone Research. 5 Questions with Sayeh Nikpay: the 340B Drug Pricing Program. Accessed July 21, 2024. https://www.cornerstone.com/insights/articles/5-questions-with-sayeh-nikpay-340b-drug-pricing-program/

[19] Maine Legislature. Title 22, §1728: prescription drug transparency report. Accessed July 21, 2024. https://legislature.maine.gov/statutes/22/title22sec1728.html

[20] Minnesota Department of Health. 340B Drug Pricing Program reporting. Accessed January 26, 2024. https://www.health.state.mn.us/data/340b/index.html

[21] US Senate. SUSTAIN 340B Act. Accessed July 21, 2024. https://www.thune.senate.gov/public/_cache/files/5e99f492-7a5e-428d-a25e-f4722cfd4b38/26132C0D072A3EF9EB32FB58CFEF5819.340b-discussion-draft-explanatory-document-and-subsequent-rfi.pdf

[22] von Elm E, Altman DG, Egger M, Pocock SJ, Gøtzsche PC, Vandenbroucke JP; STROBE Initiative. Strengthening the Reporting of Observational Studies in Epidemiology (STROBE) statement: guidelines for reporting observational studies. BMJ. 2007;335(7624):806-808. doi:10.1136/bmj.39335.541782.AD 17947786 PMC2034723

[23] CPS. 3 Ways to maximize 340B program savings. September 28, 2023. Accessed July 21, 2024. https://340breport.com/?p=11705

[24] Yue D, Pourat N, Chen X, . Enabling services improve access to care, preventive services, and satisfaction among health center patients. Health Aff (Millwood). 2019;38(9):1468-1474. doi:10.1377/hlthaff.2018.05228 31479374

[25] Jiao S, Konetzka RT, Pollack HA, Huang ES. Estimating the impact of Medicaid expansion and federal funding cuts on FQHC staffing and patient capacity. Milbank Q. 2022;100(2):504-524. doi:10.1111/1468-0009.12560 35411969 PMC9205668

[26] Smith K, Padmanabhan P, Chen A, Glied S, Desai S. The impacts of the 340B Program on health care quality for low-income patients. Health Serv Res. 2023;58(5):1089-1097. Published online July 20, 2023. doi:10.1111/1475-6773.14204 37475113 PMC10480080

[27] Owsley KM, Hasnain-Wynia R, Rooks RN, Tung GJ, Mays GP, Lindrooth RC. US hospital service availability and new 340B program participation. JAMA Health Forum. 2024;5(5):e240833. doi:10.1001/jamahealthforum.2024.0833 38700853 PMC11069079

[28] Sunita M, Desai P, McWilliams J. 340B Drug Pricing Program and hospital provision of uncompensated care. Am J Managed Care. 2021;27(10):1. https://www.ajmc.com/view/340b-drug-pricing-program-and-hospital-provision-of-uncompensated-care 10.37765/ajmc.2021.88761 PMC8544813 34668672

[29] Arizona Health Care Cost Containment System. 340B FQHC 7 FQHC look-alike pharmacy billing and reimbursement requirements. Accessed July 22, 2024. https://www.azahcccs.gov/Shared/Downloads/Reporting/PerformanceMeasures/Pharmacy/Pharmacy_340BFAQsFinal3_12_2012.pdf

[30] Nikpay S. The Medicaid windfall: Medicaid expansions and the target efficiency of hospital safety-net subsidies. J Public Econ. 2022;208:104583. doi:10.1016/j.jpubeco.2021.104583

[31] GMHC. GMHC statement on proposed New York State Medicaid carve-out plan. Accessed February 20, 2024. https://www.gmhc.org/340b/

[32] New York State. FQHC rates. Accessed February 20, 2024. https://www.health.ny.gov/health_care/medicaid/rates/fqhc/fqhc_provider_id_loc_code.htm

[33] Berkeley Research Group. Federal grantee clinics and the 340B Drug Discount Program. Accessed January 26, 2024. https://www.thinkbrg.com/insights/publications/federal-grantee-clinics-340b-drug-discount-program/

[34] Health Resources & Services Administration. National Health Center Program Uniform Data System (UDS) awardee data. Accessed August 8, 2024. https://data.hrsa.gov/tools/data-reporting/program-data/national

[35] Lam MM, Grasse NJ. Funding health care for people experiencing homelessness: an examination of federally qualified health centers’ funding streams and homeless patients served (2014-2019). Int J Environ Res Public Health. 2024;21(7):853. doi:10.3390/ijerph21070853 39063432 PMC11276671

[36] Rogers HA. Litigation continues over use of contract pharmacies in 340B Drug Discount Program. Accessed July 26, 2024. https://crsreports.congress.gov/product/pdf/LSB/LSB11163

[37] National Association of Community Health Centers. 340B: a critical program for health centers. Accessed July 21, 2024. https://www.nachc.org/wp-content/uploads/2022/06/NACHC-340B-Health-Center-Report_-June-2022-.pdf

[38] Henry B. Drug pricing & challenges to hepatitis C treatment access. J Health Biomed Law. 2018;14:265-283. 30258323 PMC6152913

[39] National Association of Community Health Centers. Manufacturer restrictions on contract pharmacies. Accessed February 21, 2024. https://opus-nc-public.digitellcdn.com/uploads/nachc/redactor/f7c28bc2b8d6e670f44ac5dc12ad8bb8a2819461701eb61b96ba33cedf82e838.pdf

[40] America’s Health Centers. By the numbers. Accessed March 13, 2024. https://www.nachc.org/resource/americas-health-centers-by-the-numbers/

[41] 340B Drug Pricing Program | HRSA. Accessed September 23, 2022. https://www.hrsa.gov/opa

[42] Health Resources and Services Administration. Fact sheet: the 340B Drug Pricing Program. Accessed September 23, 2022. https://www.aha.org/fact-sheets/2020-01-28-fact-sheet-340b-drug-pricing-program

[43] Newton W. Maine enacts nation’s second 340B provider transparency law. Accessed January 26, 2024. https://340breport.com/maine-enacts-nations-second-340b-provider-transparency-law/

[44] Sand J, Morgan ZJ, Peterson LE. Addressing social determinants of health in family medicine practices. Popul Health Manag. 2024;27(1):26-33. doi:10.1089/pop.2023.0014 37903238

[45] Gifford K, Ellis E, Lashbrook A, . A view from the states: key Medicaid policy changes: results from a 50-state Medicaid budget survey for state fiscal years 2019 and 2020. Accessed February 20, 2024. https://www.kff.org/medicaid/report/a-view-from-the-states-key-medicaid-policy-changes-results-from-a-50-state-medicaid-budget-survey-for-state-fiscal-years-2019-and-2020/

