# Errors in Conflict of Interest Disclosures

**DOI:** 10.1001/jamahealthforum.2024.4268

**Published:** 2024-11-01

In the Original Investigation titled “340B Participation and Safety Net Engagement Among Federally Qualified Health Centers,”^1^ published online on October 4, 2024, there was an error in the Conflict of Interest Disclosures. It was incorrectly stated that the fifth author received research support from Amgen; the fifth author did not receive research support from Amgen, and this disclosure has been removed. This article was corrected online.
