# Supplementary material for: 340B Participation and Safety Net Engagement Among Federally Qualified Health Centers
Source: JAMA Health Forum. 2024 Oct 4;5(10):e243360. doi: 10.1001/jamahealthforum.2024.3360 (PMC11452821; doi:10.1001/jamahealthforum.2024.3360)
Supplement: Supplement 1. — eMethods 1. Details on OPAIS and UDS cleaning and linkages eMethods 2. Association between Lagged 340B Locations and 340B Revenue eMethods 3. Regression specification eMethods 4. Robustness check using contract pharmacies as the independent variable eMethods 5. Cost of Additional Service Provision eMethods 6. Alternative specifications eTable 1. Characteristics of FQHCs reporting 340B Revenue eTable 2. Detailed Description of Independent and Dependent Variable Creation eTable 3. Regression Results for Figure 2 eTable 4. Regression results for Figure 3 eTable 5. Regression Results with outcomes in levels (not log transformed) eFigure 1. Unadjusted Relationship between 340B Locations and Outcomes eFigure 2. Average Number of 340B Registered Locations per FQHC, 2004-2021 [file jamahealthforum-e243360-s001.pdf]

Supplemental Online Content

Watts E, McGlave C, Quinones N, Bruno JP, Nikpay S. 340B participation and safety net engagement among federally qualified health centers. *JAMA Health Forum*. 2024;5(10):e243360. doi:10.1001/jamahealthforum.2024.3360

eMethods 1. Details on OPAIS and UDS cleaning and linkages
eMethods 2. Association between Lagged 340B Locations and 340B Revenue
eMethods 3. Regression specification
eMethods 4. Robustness check using contract pharmacies as the independent variable
eMethods 5. Cost of Additional Service Provision
eMethods 6. Alternative specifications
eTable 1. Characteristics of FQHCs reporting 340B Revenue
eTable 2. Detailed Description of Independent and Dependent Variable Creation
eTable 3. Regression Results for Figure 2
eTable 4. Regression results for Figure 3
eTable 5. Regression Results with outcomes in levels (not log transformed)
eFigure 1. Unadjusted Relationship between 340B Locations and Outcomes
eFigure 2. Average Number of 340B Registered Locations per FQHC, 2004-2021

This supplemental material has been provided by the authors to give readers additional information about their work.

© 2024 Watts E et al. *JAMA Health Forum.*

eMethods 1: Details on OPAIS and UDS cleaning and linkages

Data to replicate this analysis is publicly available. The Uniform Data System (UDS) can be downloaded from HRSA. Registration data for 340B can be downloaded from OPAIS.

To generate the key explanatory variable for the analysis (number of 340B registered locations and contracts per FQHC per year), we downloaded data from two databases on the Office of Pharmacy Affairs Information System:

1. Covered entities (340B registrations for clinics, called child sites, and on-site pharmacies at covered entities)
2. Contract pharmacies (340B contracts between covered entities and retail pharmacies)

The covered entity data are at the site level, including either clinics through which drugs are administered or on-site pharmacies where prescribed drugs are dispensed. The contract pharmacy data are at the level of contracts between FQHCs and individual pharmacies. Both datasets include the 340B ID number, name and address of the covered entity and the child site, on-site pharmacy, or contract pharmacy. The data also include start and termination dates of each site's registration in the 340B program.

The following section describes how we create the locations variable across the two datasets in OPAIS.

Child Sites and On-Site Pharmacies (1)
We limited the Covered Entity data to covered entities classified as "CH" (HRSA-Funded Health Center), which includes FQHCs. The Covered Entity database contains each FQHC's Grant Number, which is the same identifier used in the Uniform Data System (our primary data source for FQHC outcomes). We identified unique locations (clinics and on-site pharmacies) for each FQHC in each year by counting the number of active locations, defined by the start and termination dates. We then reshaped the FQHC grant number-level dataset to the FQHC grant number by year level datasets to create a panel with the number of locations per year. We linked these data to the UDS using the FQHC's Grant Number and year.

Contract Pharmacies (2)
As with the first dataset, we limited the contract pharmacy database to the "CH" entity type. Unlike the covered entity dataset, the contract pharmacy dataset does not contain the FQHC Grant Number. However, it does contain the covered entity's 340B ID, which also appears in the Covered Entity dataset. We created a crosswalk between the two datasets using the 340B IDs and FQHC Grant Numbers. Once the FQHC Grant Number was linked to the pharmacy contract dataset, we applied the same counting procedure for all contract pharmacies between FQHCs and pharmacies. We collapsed to the FQHC Grant Number level and re-shaped the data to create a panel dataset of the number of active locations by FQHC and year. We then linked this contract pharmacy panel to the UDS.

The key explanatory variable was generated by horizontally summing the number of locations from the covered entity dataset (on-site pharmacies and child site clinics) and the contract pharmacy dataset (340B contract pharmacies). Some FQHCs established contracts between the grantee parent site and area pharmacies and others formed contracts between child sites (sub-grantee sites) and area pharmacies. To avoid double counting contracts between grantees and contract pharmacies, we aggregated unique FQHC-pharmacy contracts to the grantee level. There were a small number of outliers in the number of pharmacy contracts. This likely stemmed from inconsistencies in the OPAIS database that occasionally counts contracts between child site clinics) and retail pharmacies. We visually examined samples of the data to visually inspect that contracts reflect relationships between the FQHC Grantee and distinct pharmacies to ensure that inconsistencies in the OPAIS data were limited. We removed remaining outliers by winsorizing the top 5% of 340B contracts for each year.

© 2024 Watts E et al. *JAMA Health Forum.*

eMethods 2: Association between Lagged 340B Locations and 340B Revenue

FQHC cost reports source significantly undercount 340B revenue. Not all 340B-participating FQHCs made the adjustments required in the cost report instructions, and those that reported did not report every year. Of the 1468 FQHCs in our dataset, 317 reported 340B revenue in at least one year between 2015 and 2020. This non-random sample of FQHCs ever reporting 340B revenue was more likely to serve areas with higher English proficiency and lower uninsurance rates relative to those that did not report (eTable 1). While these data can help provide a qualitative sense of whether our identifying assumption could be credible, we treat these estimates as merely suggestive rather than definitive estimates. The regression specification and output for this association is below:

$$340BRevenue_{fst} = \alpha_0 + \alpha_1 SITES_{f(t-1)} + \Sigma_t G + \Sigma_f C + \alpha_2 MEDICAID_{st} + \epsilon_{ft}$$

Regression results

VARIABLES	340B Revenue in $ Millions
Lagged 340B locations	0.011***
	(0.003 - 0.019)
Medicaid expansion	-0.022
	(-0.667 - 0.623)
Constant	-0.868***
	(-1.368 - -0.369)
Observations	829
R-squared	0.094
N	317
Fixed Effects	YES

An additional site is associated with a 1.1% (CI: 0.3%-1.9% increase in 340B revenue the following year between 2015 and 2020. As the median annual increase in locations for FQHCs was 1.8 locations per year between 2015 and 2020, our estimates imply a 2.0% (1.8 locations*1.1%) increase in 340B revenue per year for the median FQHC. Scaled by the 340B revenues for the median health center in 2015, the first year for which we have 340B revenue per year (2015 median 340B revenue of $739,472*2.0%) for the median FQHC.

© 2024 Watts E et al. *JAMA Health Forum.*

eMethods 3: Regression specification

We estimated ordinary least squares regressions with measures of safety-net engagement as the dependent variables and the lagged number of 340B-registered locations, FQHC fixed effects, time fixed effects, and a time-varying Medicaid expansion indicator.

$$Y_{fst} = \alpha_0 + \alpha_1 LOC_{f(t-1)} + \Sigma_t G + \Sigma_f C + \alpha_2 MEDICAID_{st} + \epsilon_{ft}$$

Here, Y is a set of outcome measures such as 340B revenue or measures of safety-net engagement, which vary at the FQHC-level, f, and the year level, t. All outcomes are logged to account for non-linearities in the data LOC is a measure of 340B program size in each year based on the number of 340B-registered child sites and contract pharmacies from which drugs can be dispensed. We lag this variable to account for time for FQHCs to make decisions and invest in strategies after 340B revenue is generated. The vector G represents year-level fixed effects to account for common changes to the funding environment, and the variable MEDICAID is an indicator set to 1 if the FQHC is located in a state that has expanded Medicaid. The vector C represents FQHC-level fixed effects to account for time-invariant differences between FQHCs. We estimate the equation using Ordinary Least Squares and cluster standard errors at the FQHC level. If FQHCs expand safety-net services, we expect α to be positive for visits and services. Specifically, we expect α to be larger for uninsured and publicly insured patients than privately insured patients.

© 2024 Watts E et al. *JAMA Health Forum.*

eMethods 4: Robustness check using contract pharmacies as the independent variable

Since the additional 340B-registrations at child sites may be associated with increased patients served and services rendered, we tested the robustness of our primary results using lagged contract pharmacies as the independent variable. This approach avoids potential confounding between the lagged number of 340B registered locations—which includes 340B-registered child sites—and services offered because the UDS does not count services rendered at contract pharmacies. Contract pharmacies comprise 72% of 340B-registered locations in the primary specification of the independent variable. The bar chart below shows similar growth in 340B participation over time.

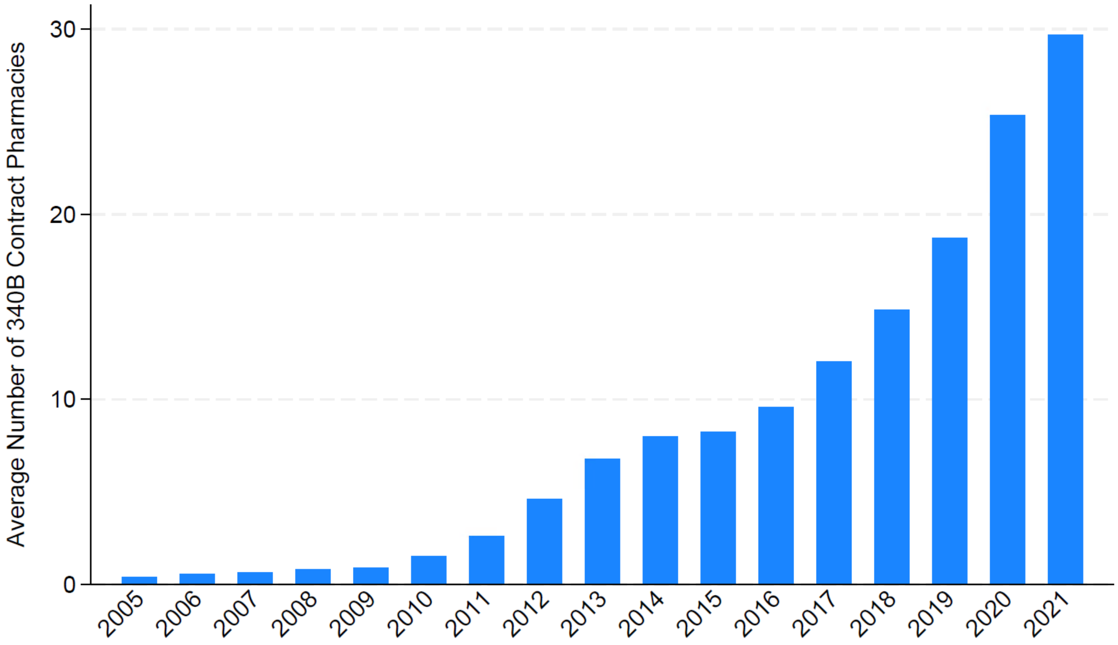

eMethods Figure 1. Average 340B Contract Pharmacies, 2005-2021
Source: Uniform Data System merged to 340B Office of Pharmacy Affairs Database, 2005-2021 (N=599 in 2004; N=1,341 in 2021). Note: The figure shows the average number of on-site 340B contract pharmacies per FQHC per year.

The alternate specification is identical to the primary model apart from the independent variable. We estimated ordinary least squares regressions with measures of safety-net engagement as the dependent variables and the lagged number of 340B contract pharmacies (CP) winsorized at 5%, FQHC fixed effects, time fixed effects, and a time-varying Medicaid expansion indicator.

$$Y_{fst} = \alpha_0 + \alpha_1 CP_{f(t-1)} + \Sigma_t G + \Sigma_f C + \alpha_2 MEDICAID_{st} + \epsilon_{ft}$$

Results from this specification are nearly identical in size and direction to the primary results. We see small, but statistically significant associations between growth in contract pharmacies in the previous year and outcomes. The following graphs show the magnitude and confidence intervals between the independent variable (lagged 340B contract pharmacies) and the outcomes of interest.

© 2024 Watts E et al. *JAMA Health Forum.*

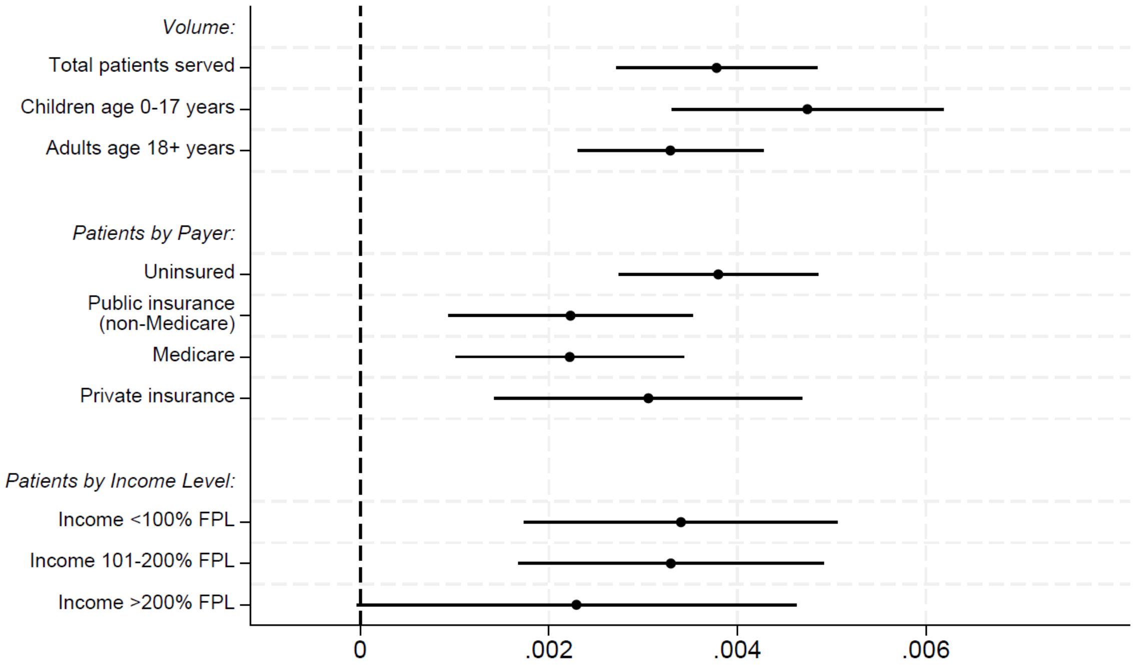

eMethods 4, Figure 2: Association of Lagged Contract Pharmacies with Volume and Payer Mix, 2005-2022 Source: Uniform Data System (2005-2022) linked to Office of Pharmacy Affairs Information System (2023). Note: Magnitude and 95% confidence intervals of associations between lagged 340B contract pharmacies and outcomes of interest (volume, patients by payer, and patients by income level). All outcomes are log-transformed.

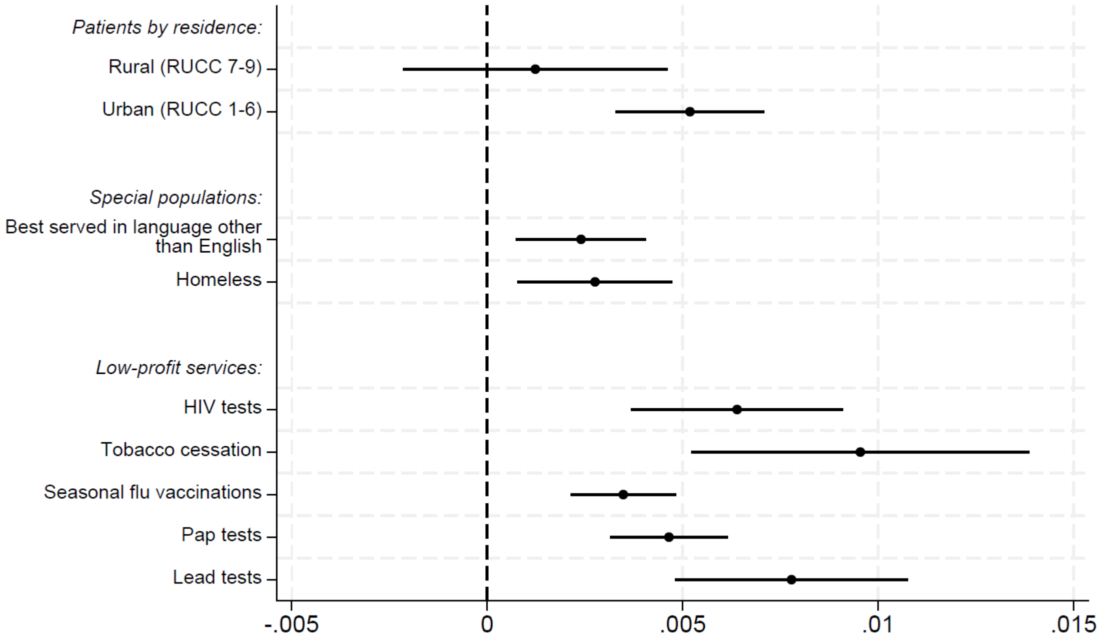

eMethods 4, Figure 3: Association of Lagged Contract Pharmacies with Patient Populations and Services, 2005-2022. Source: Uniform Data System (2005-2022) linked to 340B Outpatient Pharmacy Information System (2023). Note: Magnitude and 95% confidence intervals of associations between lagged 340B contract pharmacies and

© 2024 Watts E et al. *JAMA Health Forum.*

outcomes of interest (patient residence, special populations, and low-profit services). All outcomes are log-transformed.

Results for patient volume and services offered are nearly identical to the primary specification, which allays potential concerns about confounding between 340-registered locations that generate revenue and offer services included in our outcomes. Tables with regression results are below.

© 2024 Watts E et al. *JAMA Health Forum.*

eMethods 4, Table 1: Numerical results for eMethods 4, Figure 2.

	(1)	(2)	(3)	(4)	(5)	(6)	(7)	(8)	(9)	(10)	(11)
VARIABLES	340B Revenue in $ Millions	Total patients served	Children age 0-17 years	Adults age 18+ years	Uninsured	Public insurance (non-Medicare)	Medicare	Private insurance	Income <100% FPL	Income 101-200% FPL	Income >200% FPL
Lagged 340B Contract Pharmacies (w5)	0.013**	0.004***	0.005***	0.003***	0.004***	0.002***	0.002***	0.003***	0.003***	0.003***	0.002*
	(0.003 - 0.024)	(0.003 - 0.005)	(0.003 - 0.006)	(0.002 - 0.004)	(0.003 - 0.005)	(0.001 - 0.004)	(0.001 - 0.003)	(0.001 - 0.005)	(0.002 - 0.005)	(0.002 - 0.005)	(-0.000 - 0.005)
Medicaid expansion	-0.015	0.014	0.002	0.010	-0.421***	0.193***	0.102***	-0.240***	-0.113***	0.028	0.047
	(-0.657 - 0.627)	(-0.038 - 0.066)	(-0.069 - 0.072)	(-0.037 - 0.057)	(-0.473 - - 0.369)	(0.129 - 0.258)	(0.040 - 0.165)	(-0.327 - - 0.153)	(-0.180 - - 0.046)	(-0.051 - 0.106)	(-0.072 - 0.166)
Constant	-0.859***	7.617***	7.702***	8.544***	7.952***	7.913***	6.141***	6.684***	8.076***	6.825***	5.512***
	(-1.355 - - 0.364)	(7.536 - 7.699)	(7.661 - 7.744)	(8.512 - 8.577)	(7.913 - 7.991)	(7.872 - 7.954)	(6.094 - 6.188)	(6.627 - 6.742)	(8.027 - 8.125)	(6.764 - 6.886)	(5.421 - 5.603)
Observations	829	21,527	21,418	21,521	21,526	21,486	21,299	21,200	21,478	21,321	20,783
R-squared	0.098	0.514	0.073	0.314	0.141	0.406	0.375	0.297	0.070	0.096	0.111
Number of id	317	1,468	1,466	1,468	1,467	1,468	1,463	1,465	1,468	1,468	1,463
Fixed Effects	YES	YES	YES	YES	YES	YES	YES	YES	YES	YES	YES

Notes: Uniform Data System merged to 340B Office of Pharmacy Affairs Database. Robust confidence intervals in parentheses. All standard errors are clustered at the Federally qualified health center level. Medicaid expansion indicator omitted from the earliest period by default. Fixed effects included for year and FQHC. Star levels indicate significance at the 1%, 5%, and 10% levels.

© 2024 Watts E et al. *JAMA Health Forum.*

eMethods 4, Table 2: Numerical results for eMethods 4, Figure 3.

VARIABLES	(1)<br>Best served in language other than English	(2)<br>Homeless	(3)<br>Rural (RUCC 7-9)	(4)<br>Urban (RUCC 1-6)	(5)<br>HIV tests	(6)<br>Tobacco cessation	(7)<br>Seasonal flu vaccinations	(8)<br>Pap tests	(9)<br>Lead tests
Lagged 340B Contract Pharmacies (w5)	0.002***	0.003***	0.001	0.005***	0.006***	0.010***	0.003***	0.005***	0.008***
	(0.001 - 0.004)	(0.001 - 0.005)	(-0.002 - 0.005)	(0.003 - 0.007)	(0.004 - 0.009)	(0.005 - 0.014)	(0.002 - 0.005)	(0.003 - 0.006)	(0.005 - 0.011)
Medicaid expansion	-0.001	-0.023	-0.063	-0.034	0.004	-0.136	0.019	-0.104***	0.108
	(-0.091 - 0.089)	(-0.148 - 0.102)	(-0.173 - 0.048)	(-0.117 - 0.050)	(-0.125 - 0.133)	(-0.361 - 0.088)	(-0.051 - 0.088)	(-0.177 - -0.030)	(-0.038 - 0.253)
Constant	6.099***	5.985***	4.440***	7.162***	4.793***	3.628***	6.554***	6.357***	4.163***
	(6.026 - 6.171)	(5.683 - 6.288)	(4.321 - 4.559)	(7.049 - 7.274)	(4.681 - 4.905)	(3.377 - 3.878)	(6.494 - 6.614)	(6.296 - 6.418)	(4.042 - 4.284)
Observations	17,886	16,323	10,732	19,817	17,449	11,947	17,345	21,329	12,829
R-squared	0.169	0.113	0.235	0.244	0.216	0.119	0.132	0.035	0.070
Number of id	1,465	1,436	1,184	1,465	1,443	1,420	1,457	1,465	1,351
Fixed Effects	YES	YES	YES	YES	YES	YES	YES	YES	YES

Notes: Uniform Data System merged to 340B Office of Pharmacy Affairs Database. Robust confidence intervals in parentheses. All standard errors are clustered at the Federally qualified health center level. Medicaid expansion indicator omitted from the earliest period by default. Fixed effects included for year and FQHC. Star levels indicate significance at the 1%, 5%, and 10% levels.

© 2024 Watts E et al. *JAMA Health Forum.*

eMethods 5: Cost of Additional Service Provision

We estimated the cost of additional uncompensated and undercompensated care provided by the median FQHC that reported 340B revenue. We used the median values of patient volume from 2020 to align with the last year for which we had data on 340B revenue, which is reported in the main text, and estimated to be $17,600 per 340B location. The cost per patient is from the average "Total Accrued Cost per Patient" from the 2020 UDS reported by HRSA. We assume that care provided to uninsured patients is uncompensated and conservatively estimate that the cost of serving an uninsured patient is equivalent to the average across all patients. The cost of serving patients needing enabling services (unhoused patients, patients needing translation services) likely exceeds the average cost of serving a patient. We refrain from summing the number of additional uninsured patients served with the number of patients needing enabling services because there may be overlap in these groups.

eMethods 5, Table 1. Estimated cost of additional uncompensated and undercompensated care per 340B location increase

	Median among FQHCs that reported 340B revenue (n=314)	% Increase (regression results)	Increase	Cost per patient	**Total cost**
Uninsured patients served	2554	0.4%	10	$1,156.82[1]	**$11,568.20**
Unhoused patients served	212	0.3%	1	>$1,156.82[1,2]	**>$1,156.82**
Patients best served in a language other than English	1167	0.3%	4	>$1,156.82[1]	**>$4,627.28**

For added context, we estimated the cost of increased low-profit service provision per additional 340B location for the median FQHC. Cost methodology is in Table 2 below.

eMethods 5, Table 2. Estimated cost of low-profit services per 340B location increase

	Median among FQHCs that reported 340B revenue (n=314)	% Increase (regression results)	Increase (number of tests/visits)	Estimated Cost	Cost methodology	Total cost
HIV tests	507	0.7%	4	$173.50[3]	Medicare PPS rate for Evaluation/Management office visit [Assumption]	$694.00
Tobacco cessation visits	203	1.0%	2	$219.16[4]	Reimbursement for intermediate tobacco cessation visit for existing patient	$374.38
Seasonal flu vaccinations	1740	0.4%	7	$30.00[5]	Physician Fee Schedule*	$210.00
Pap tests	538	0.5%	3	$173.50[3]	Medicare PPS rate for Evaluation/Management office visit	$520.50

© 2024 Watts E et al. *JAMA Health Forum.*

Lead tests	85	0.8%	1	$173.50[3]	Medicare PPS rate for Evaluation/Management office visit [Assumption]	$173.50
**Total**						**$1,972.38**

*Note: The reimbursement rate for influenza vaccines is based on calendar year 2023.

References:

1. Health Resources & Services Administration. National Health Center Program Uniform Data System (UDS) Awardee Data. https://data.hrsa.gov/tools/data-reporting/program-data/national. Accessed August 1, 2024

2. Lam, M. M., & Grasse, N. J. (2024). Funding Health Care for People Experiencing Homelessness: An Examination of Federally Qualified Health Centers' Funding Streams and Homeless Patients Served (2014–2019). International Journal of Environmental Research and Public Health, 21(7), 853–853. https://doi.org/10.3390/ijerph21070853

3. CMS Medicare Learning Network. Update to the Federally Qualified Health Center (FQHC) Prospective Payment System (PPS) for Calendar Year (CY) 2020 - Recurring File Update. https://www.cms.gov/files/document/mm11500.pdf. Accessed August 1, 2024

4. National Association of Community Health Centers. Reimbursement Tips: FQHC Requirements for Tobacco Cessation Counseling. chrome-extension://efaidnbmnnnibpcajpcglclefindmkaj/https://www.nachc.org/wp-content/uploads/2023/07/Reimbursement-Tips_Tobacco-Cessation.pdf. Accessed August 1, 2024.

5. CMS Medicare Learning Network. National Fee Schedule for Medicare Part B Vaccine Administration. chrome-extension://efaidnbmnnnibpcajpcglclefindmkaj/https://www.cms.gov/files/document/mm12943-national-fee-schedule-medicare-part-b-vaccine-administration.pdf . Accessed August 1, 2024.

© 2024 Watts E et al. *JAMA Health Forum.*

eMethods 6: Alternative specifications

The primary results show associations (not causal relationships) between lagged increases in 340B locations and outcomes. We explored alternative study designs using instrumental variables to try and capture a causal relationship between 340B participation and safety-net care provision, but the methods were not predictive. Below is a summary of the alternative specifications with explanations of their limitations.

Approach Details	Limitations
We calculated the distance between an FQHC and all retail pharmacies in 2010. We attempted to use the 2010 expansion of contract pharmacies and an indicator for having a retail pharmacy within one, two and five miles as an instrument for an FQHC's potential to benefit from the 2010 regulation.	The distance between FQHCs and pharmacies The instrument had low predictive value for adding contract pharmacies after 2010. Additionally, approximately 90% of the sample of FQHCs had at least one retail pharmacy within one mile, leading to an unbalanced sample. The FQHCs with pharmacies nearby were more likely to be located in urban areas, which led to bias in the analysis.
We used variation in Medicaid financing policy related to 340B and FQHCs. Several states (Arizona, Minnesota) chose to prevent FQHCs from charging states more than the 340B acquisition cost for Medicaid patients. These policies essentially prevent FQHCs from generating 340B revenue from Medicaid patients.	The introduction of these prohibitions in specific states occurred at the same time as the ACA and/or pandemic-related Medicaid expansions, leading to confounding.

© 2024 Watts E et al. *JAMA Health Forum.*

eTable 1: Characteristics of FQHCs reporting 340B Revenue

	Revenue not reported			Reports 340B revenue			
	N	Mean	SD	N	Mean	SD	Diff
Funding sources							
% Receives Community Health Center (CHC) funding	1029	93.10	25.36	316	95.89	19.89	3.526***
% Receives Migrant Health Center (MHC) funding	1029	11.76	32.23	316	14.56	35.32	3.085
% Receives Healthcare for the Homeless (HO) funding	1029	21.57	41.15	316	21.52	41.16	-1.248
% Receives Public Housing (PH) funding	1029	6.90	25.36	316	8.23	27.52	0.866
Demographics of area served (American Community Survey)							
% population under 1.37 of the poverty threshold	1021	29.92	8.41	315	30.13	7.47	0.321
% population below 1.99 of the poverty threshold	1021	43.27	9.72	315	43.49	8.59	0.261
% civilian labor force that is unemployed (ages 16 and over)	1021	10.06	3.47	315	10.07	3.07	0.057
% limited English speaking households	1021	5.90	6.61	315	4.95	5.87	-0.914**
% population with less than high school education (ages 25 and over)	1021	17.90	7.72	315	17.17	6.70	-0.547
% population with a disability	1021	14.58	4.05	315	15.21	3.98	0.615**
% population under 64 yrs with no health insurance coverage	1021	17.95	6.70	315	17.20	5.34	-0.613*
Outcomes of interest							
Total patients served	1028	17,304.69	20,807.88	316	19,210.95	24,714.71	2,522.823*
Children age 0-17 years	1029	5,378.06	7,745.65	316	5,715.36	8,667.46	579.273
Adults age 18+ years	1029	11,804.28	13,533.83	316	13,344.19	16,539.29	1,945.790*
Uninsured	1029	4,271.20	5,395.59	316	4,550.66	6,455.10	424.969
Public insurance (non-Medicare)	1029	10,656.61	14,724.86	316	11,602.09	17,010.42	1,364.762
Medicare	1024	1,545.31	2,166.30	316	1,711.20	2,189.76	290.234**
Private insurance	1029	2,834.16	3,835.88	316	3,499.63	4,710.61	802.787***
Income less than 100 pct FPL	1026	9,049.13	13,248.83	316	9,630.28	15,788.76	1,276.962
Income 101-200 pct FPL	1029	2,724.40	4,007.16	316	2,948.86	5,173.11	241.077
Income greater than 200 pct FPL	1026	982.40	2,134.38	314	1,161.20	2,471.14	212.181
Best served in language other than English	1021	3,900.87	7,800.78	312	3,781.39	9,310.79	-21.376
Homeless	997	928.70	2,281.52	304	850.50	2,220.23	-74.571
Rural (RUCC 7-9)	1022	1,048.02	2,998.41	316	1,480.32	3,916.01	469.095*
Urban (RUCC 1-6)	1022	16,358.22	20,938.04	316	17,730.63	24,835.73	2,013.138

© 2024 Watts E et al. *JAMA Health Forum.*

	Revenue not reported			Reports 340B revenue			
	N	Mean	SD	N	Mean	SD	Diff
HIV tests	1029	954.71	2,562.35	316	1,408.08	4,287.68	367.607*
Tobacco cessation	1029	1,006.09	3,352.94	316	1,341.59	5,116.63	411.169
Seasonal flu vaccinations	1029	2,569.49	4,581.59	316	2,761.13	4,836.28	230.492
Pap tests	1029	1,402.57	2,092.07	316	1,649.72	2,730.87	201.846
Lead tests	1029	328.84	876.79	316	401.56	1,413.21	75.868

Notes: Table shows averages and standard deviations for 2015, the first year of available data for 340B revenue. The Diff column is the coefficient of a simple regression of reporting status on the variable, with clustered standard errors at the FQHC level. Stars indicate statistical significance at 1, 5 and 10-percent levels.

© 2024 Watts E et al. *JAMA Health Forum.*

eTable 2: Detailed Description of Independent and Dependent Variable Creation

The following exhibit provides details on how variables were created and in addition, the years over which the variables are reported.

**Variable**	**Notes**	**Data source**	**N**	**Years available**
Registered 340B locations	Sum of on-site 340B locations and retail pharmacy contracts	HRSA Office of Pharmacy Affairs Information System	20,221	Entire period
340B revenue	Revenue from 340B program (net savings)	FQHC CMS Cost reports and UDS	832	2015-2020
Patient volume	Annual number of patients served by FQHC (total, adults age >18 years, children aged 0-17 years)	UDS	21,544	Entire period
Payer mix	Number of patients by insurer type (uninsured, publicly insured, privately insured, Medicare)	UDS	21,561	Entire period
Income mix	Number of patients by income level (under 100%, 101-200%, and over 200% of federal poverty line)	UDS	21,561	Entire period
Special populations				
Patients best served in a language other than English	Number of patients	UDS	18,387	2008-2022
Unhoused patients	Number of patients	UDS	20,121	Entire period
Number of patients living in rural zip codes	Calculated by authors based on number of patients by residential zip codes classified as RUCC >6	UDS, USDA Rural-urban Continuum Codes 2013	20,185	2005-2021
Low-profit services				
HIV tests	Number of visits by diagnosis	UDS	21,158	Entire period
Pap tests	Number of visits by diagnosis	UDS	21,511	Entire period
Flu vaccinations	Number of visits by diagnosis	UDS	17,585	2009-2022
Serum lead tests	Number of visits by diagnosis	UDS	17,110	2009-2022
Tobacco cessation	Number of visits by diagnosis	UDS	17,110	2009-2022

© 2024 Watts E et al. *JAMA Health Forum.*

eTable 3: Regression Results for Figure 2

VARIABLES	Total patients served	Children age 0-17 years	Adults age 18+ years	Uninsured	Public insurance (non-Medicare)	Medicare	Private insurance	Income <100% FPL	Income 101-200% FPL	Income >200% FPL
Lagged 340B locations	0.004*** (0.003 - 0.005)	0.005*** (0.004 - 0.007)	0.004*** (0.003 - 0.005)	0.004*** (0.003 - 0.005)	0.003*** (0.002 - 0.004)	0.003*** (0.002 - 0.004)	0.004*** (0.002 - 0.005)	0.004*** (0.002 - 0.006)	0.004*** (0.002 - 0.006)	0.003*** (0.001 - 0.005)
Medicaid expansion	0.014 (-0.038 - 0.066)	0.002 (-0.068 - 0.071)	0.010 (-0.037 - 0.056)	-0.420*** (-0.472 - - 0.369)	0.193*** (0.129 - 0.257)	0.102*** (0.040 - 0.164)	0.240*** (-0.326 - - 0.153)	0.113*** (-0.180 - - 0.046)	0.027 (-0.051 - 0.106)	0.046 (-0.072 - 0.165)
Constant	7.613*** (7.532 - 7.694)	7.697*** (7.656 - 7.738)	8.541*** (8.508 - 8.573)	7.948*** (7.909 - 7.987)	7.911*** (7.870 - 7.952)	6.139*** (6.092 - 6.186)	6.681*** (6.624 - 6.739)	8.073*** (8.024 - 8.122)	6.821*** (6.761 - 6.882)	5.510*** (5.419 - 5.601)
Observations	21,527	21,418	21,521	21,526	21,486	21,299	21,200	21,478	21,321	20,783
R-squared	0.516	0.079	0.319	0.145	0.408	0.376	0.299	0.072	0.098	0.112
N	1,468	1,466	1,468	1,467	1,468	1,463	1,465	1,468	1,468	1,463
Fixed Effects	YES	YES	YES	YES	YES	YES	YES	YES	YES	YES

Notes: Uniform Data System merged to 340B Office of Pharmacy Affairs Database and Federally Qualified Health Center Cost Reports, 2015-2020. All outcomes are logged. Robust confidence intervals in parentheses. All standard errors are clustered at the Federally qualified health center level. Fixed effects included for year and FQHC. Star levels indicate significance at the 1%, 5%, and 10% levels.

© 2024 Watts E et al. *JAMA Health Forum.*

eTable 4: Regression results for Figure 3

Variables	Best served in language other than English	Homeless	Rural (RUCC 7-9)	Urban (RUCC 1-6)	HIV tests	Tobacco cessation	Seasonal flu vaccinations	Pap tests	Lead tests
Lagged 340B locations	0.003*** (0.001 - 0.005)	0.003*** (0.001 - 0.005)	0.002 (-0.001 - 0.005)	0.006*** (0.004 - 0.008)	0.007*** (0.004 - 0.009)	0.010*** (0.005 - 0.014)	0.004*** (0.003 - 0.005)	0.005*** (0.004 - 0.007)	0.008*** (0.006 - 0.011)
Medicaid expansion	-0.001 (-0.090 - 0.088)	-0.023 (-0.148 - 0.101)	-0.062 (-0.173 - 0.048)	-0.034 (-0.118 - 0.049)	0.004 (-0.125 - 0.133)	-0.135 (-0.359 - 0.089)	0.019 (-0.050 - 0.088)	-0.103*** (-0.176 - - 0.030)	0.108 (-0.037 - 0.253)
Constant	6.095*** (6.022 - 6.167)	5.986*** (5.684 - 6.288)	4.437*** (4.318 - 4.556)	7.156*** (7.044 - 7.268)	4.787*** (4.675 - 4.899)	3.613*** (3.362 - 3.864)	6.548*** (6.488 - 6.608)	6.352*** (6.291 - 6.413)	4.148*** (4.026 - 4.269)
Observations	17,886	16,323	10,732	19,817	17,449	11,947	17,345	21,329	12,829
R-squared	0.170	0.114	0.236	0.245	0.217	0.120	0.134	0.039	0.072
N	1,465	1,436	1,184	1,465	1,443	1,420	1,457	1,465	1,351
Fixed Effects	YES	YES	YES	YES	YES	YES	YES	YES	YES

Notes: Uniform Data System merged to 340B Office of Pharmacy Affairs Database and Federally Qualified Health Center Cost Reports, 2015-2020. All outcomes are logged. Robust confidence intervals in parentheses. All standard errors are clustered at the Federally qualified health center level. Fixed effects included for year and FQHC. Star levels indicate significance at the 1%, 5%, and 10% levels.

© 2024 Watts E et al. *JAMA Health Forum.*

eTable 5: Regression Results with outcomes in levels (not log transformed)

Panel A: Figure 2 Outcomes

Variables	340B Revenue in $ Millions	Total patients served	Children age 0-17 years	Adults age 18+ years	Uninsured	Public insurance (non-Medicare)	Medicare	Private insurance	Income <100% FPL	Income 101-200% FPL	Income >200% FPL
Lagged 340B locations	0.04*** (0.02 - 0.07)	257.28*** (210.06 - 304.49)	60.87*** (44.47 - 77.28)	168.23*** (136.12 - 200.34)	7.92 (-2.22 - 18.06)	185.39*** (149.27 - 221.52)	26.99*** (21.12 - 32.86)	51.64*** (40.27 - 63.01)	85.38*** (56.74 - 114.02)	36.12*** (26.47 - 45.76)	14.77*** (7.55 - 21.98)
Medicaid expansion	0.12 (-0.49 - 0.74)	986.73* (-99.00 - 2,072.46)	-26.53 (-452.93 - 399.86)	791.42** (81.02 - 1,501.81)	-1,774.26*** (-2,144.24 - -1,404.29)	3,360.12*** (2,634.55 - 4,085.69)	167.05** (24.00 - 310.09)	-678.37*** (-1,022.95 - -333.79)	119.59 (-542.04 - 781.22)	359.54*** (101.67 - 617.40)	87.56 (-116.55 - 291.67)
Constant	0.41 (-0.23 - 1.06)	2,062.65*** (887.49 - 3,237.81)	4,275.78*** (4,002.77 - 4,548.79)	7,219.43*** (6,740.16 - 7,698.69)	5,179.63*** (4,962.21 - 5,397.06)	4,535.98*** (4,005.71 - 5,066.25)	814.32*** (730.02 - 898.62)	1,716.27*** (1,551.24 - 1,881.30)	6,443.70*** (5,921.11 - 6,966.28)	1,937.71*** (1,766.08 - 2,109.34)	839.90*** (718.15 - 961.65)
Observations	829	21,542	21,557	21,557	21,557	21,557	21,456	21,557	21,515	21,557	21,266
R-squared	0.27	0.36	0.14	0.34	0.08	0.37	0.34	0.27	0.10	0.10	0.05
N	317	1,468	1,468	1,468	1,468	1,468	1,466	1,468	1,468	1,468	1,466
Fixed Effects	YES	YES	YES	YES	YES	YES	YES	YES	YES	YES	YES

© 2024 Watts E et al. *JAMA Health Forum.*

Panel B: Figure 3 Outcomes

Variables	Best served in language other than English	Homeless	Rural (RUCC 7-9)	Urban (RUCC 1-6)	HIV tests	Tobacco cessation	Seasonal flu vaccinations	Pap tests	Lead tests
Lagged 340B locations	53.91*** (36.13 - 71.69)	10.67*** (6.48 - 14.86)	7.38** (0.01 - 14.75)	294.69*** (239.29 - 350.09)	45.44*** (34.71 - 56.16)	68.31*** (25.53 - 111.10)	36.78*** (27.91 - 45.64)	6.93*** (2.36 - 11.49)	6.37*** (4.44 - 8.31)
Medicaid expansion	100.26 (-291.54 - 492.06)	105.78* (-19.79 - 231.34)	-116.25 (-283.67 - 51.18)	1,265.06** (229.06 - 2,301.06)	83.84 (-176.07 - 343.75)	91.59 (-293.02 - 476.19)	363.99*** (148.33 - 579.65)	-66.76 (-188.30 - 54.78)	32.99 (-17.52 - 83.50)
Constant	3,227.75*** (2,954.20 - 3,501.30)	222.13** (36.08 - 408.18)	574.89*** (460.80 - 688.98)	1,543.48*** (381.39 - 2,705.56)	268.60*** (113.69 - 423.51)	-149.33 (-429.79 - 131.14)	1,645.20*** (1,429.89 - 1,860.51)	1,410.57*** (1,318.48 - 1,502.65)	238.55*** (188.32 - 288.78)
Observations	18,385	20,119	20,185	20,185	21,156	17,277	17,583	21,509	17,108
R-squared	0.10	0.04	0.05	0.36	0.19	0.05	0.14	0.02	0.04
N	1,467	1,466	1,467	1,467	1,468	1,462	1,462	1,468	1,461
Fixed Effects	YES	YES	YES	YES	YES	YES	YES	YES	YES

Notes: Uniform Data System merged to 340B Office of Pharmacy Affairs Database. Robust confidence intervals in parentheses. All standard errors are clustered at the Federally qualified health center level. Medicaid expansion indicator omitted from the earliest period by default. Fixed effects included for year and FQHC. Star levels indicate significance at the 1%, 5%, and 10% levels.

© 2024 Watts E et al. *JAMA Health Forum.*

eFigure 1: Unadjusted Relationship between 340B Locations and Outcomes
The following presents binned scatter plots depicting the relationship between the average of each dependent variable in the analysis and equally sized bins of lagged 340B locations.

a. Outcomes from Figure 2 (volume, payer, income level)

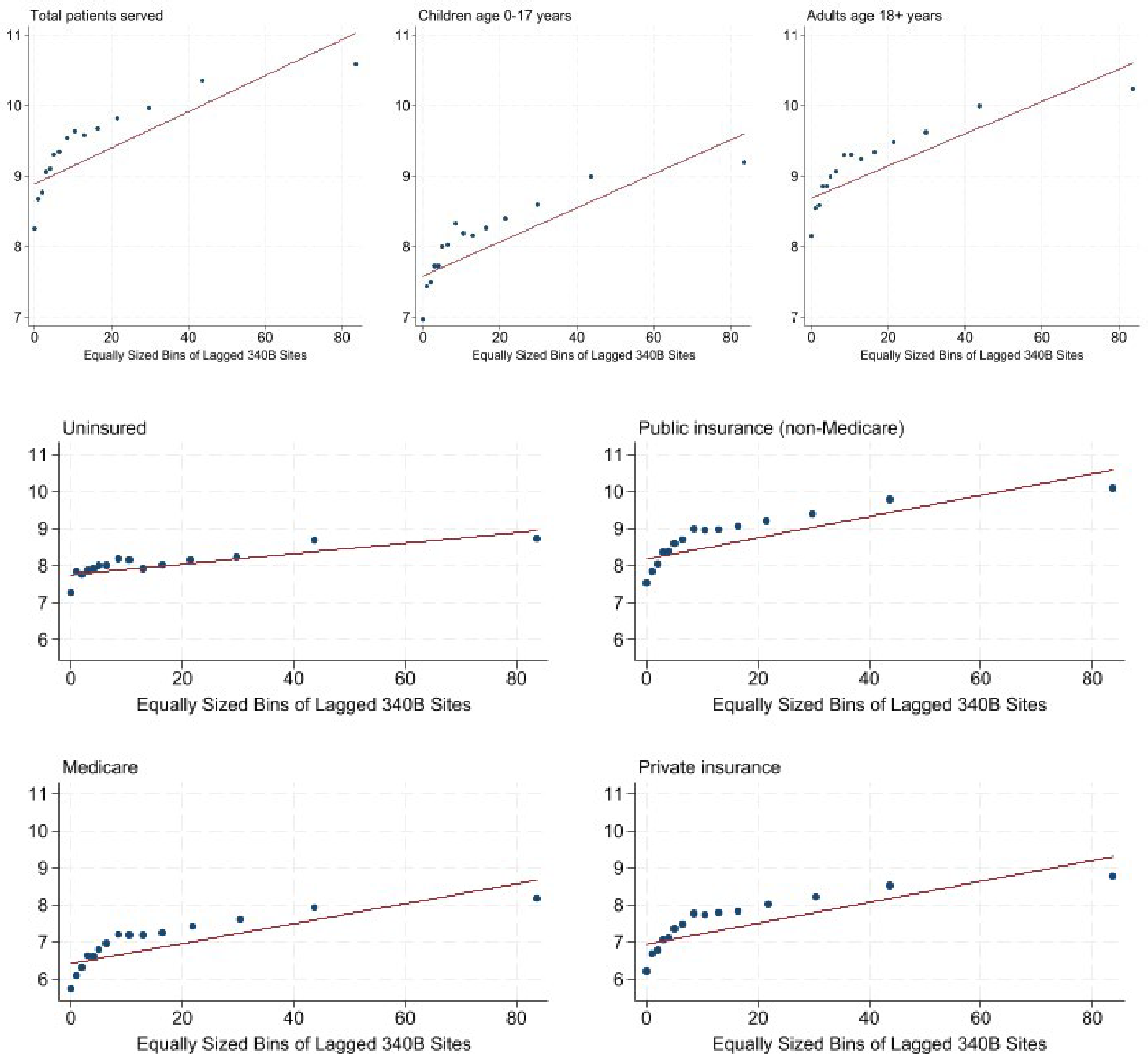

© 2024 Watts E et al. *JAMA Health Forum.*

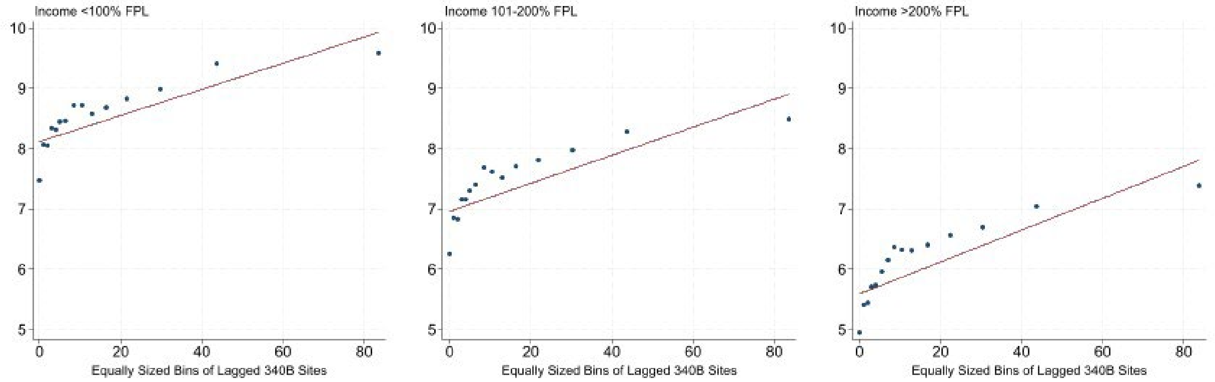

Source: Uniform Data System merged to 340B Office of Pharmacy Affairs Database and Federally Qualified Health Center Cost Reports, 2005-2022. Note: The figure shows binned scatter plots with the average of total patients, and patients by payer category: private, uninsured, Medicare, Medicaid and other public payers, and duals, against equally sized bins of the lagged number of locations. All patient measures were logged.

© 2024 Watts E et al. *JAMA Health Forum.*

b. Outcomes from Figure 3 (rural/urban, special pop, low-profit services)

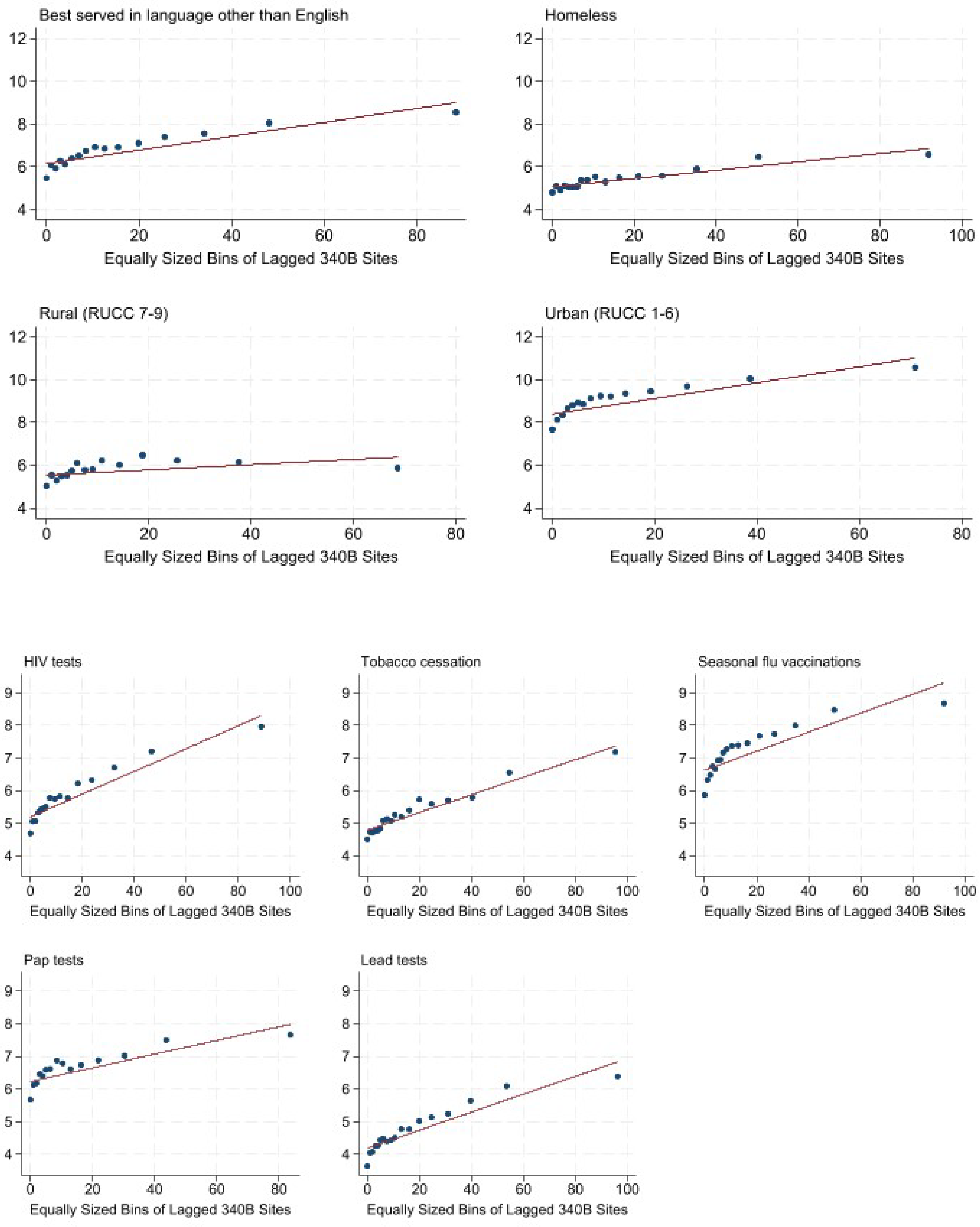

Source: Uniform Data System merged to 340B Office of Pharmacy Affairs Database and Federally Qualified Health Center Cost Reports, 2005-2022. Note: The figure shows binned scatter plots with the average of total visits by service category: HIV/AIDS sting, tobacco cessation, flu vaccinations, and Pap tests. All patient measures were logged.

© 2024 Watts E et al. *JAMA Health Forum.*

eFigure 2: Average Number of 340B Registered Locations per FQHC, 2004-2021

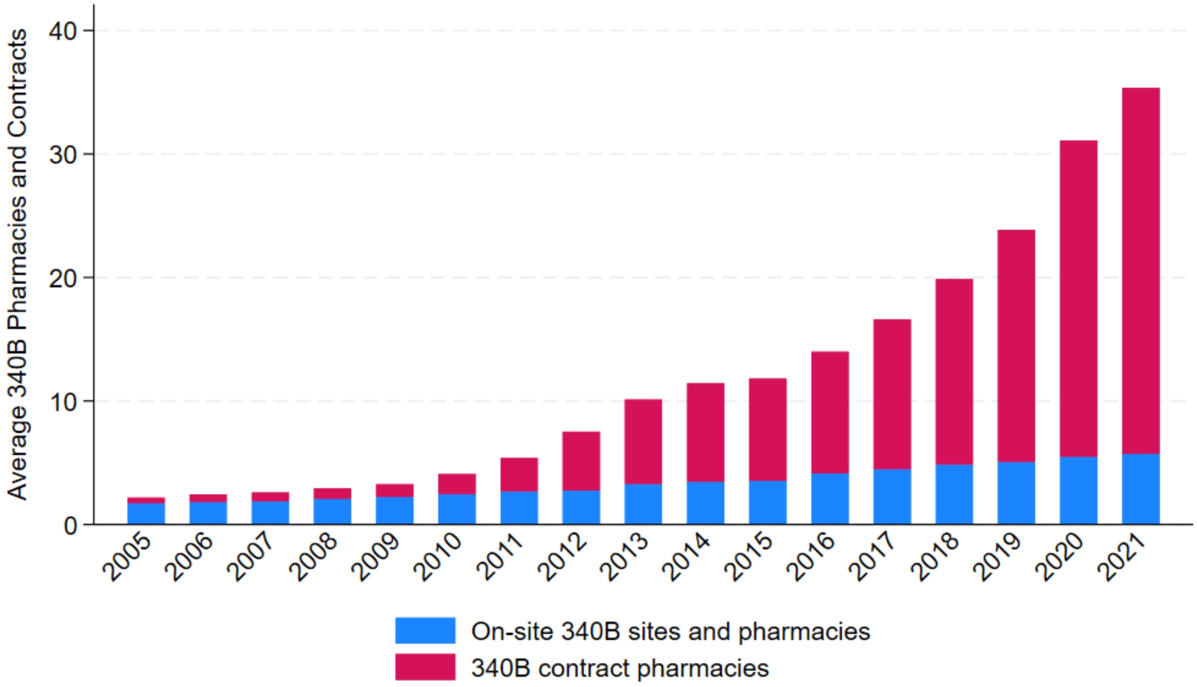

Source: Uniform Data System merged to 340B Office of Pharmacy Affairs Database, 2004-2021 (N=599 in 2004; N=1,341 in 2021). Note: The figure shows the average number of 340B-registered locations (including on-site and contract pharmacies) per FQHC per year.

© 2024 Watts E et al. *JAMA Health Forum.*
