# Supplementary material for: 340B Participation and Safety Net Engagement Among Federally Qualified Health Centers
Source: JAMA Health Forum. 2024 Oct 4;5(10):e243360. doi: 10.1001/jamahealthforum.2024.3360 (PMC11452821; doi:10.1001/jamahealthforum.2024.3360)
Supplement: Supplement 2. — Data sharing statement [file jamahealthforum-e243360-s002.pdf]

Data Sharing Statement

Watts. 340B Participation and Safety Net Engagement Among Federally Qualified Health Centers. *JAMA Health Forum*. Published October 04, 2024.
doi:10.1001/jamahealthforum.2024.3360

Data
Data available: Yes
Data types: Data (not involving human participants)
How to access data: Data to replicate this analysis is publicly available. The Uniform Data System (UDS) can be downloaded from HRSA: https://data.hrsa.gov/data/download?data=HSCD#HSCD Registration data for 340B can be downloaded from OPAIS: https://340bopais.hrsa.gov/home
When available: beginning date: 04-30-2023

Supporting Documents
Document types: None

Additional Information
Who can access the data: Anyone
Types of analyses: N/A
Mechanisms of data availability: Without investigator support
